# A new posterosuperior screw placement strategy to avoid in-out-in screws in femoral neck fractures

**DOI:** 10.3389/fsurg.2023.1142135

**Published:** 2023-03-17

**Authors:** Shi-Jie Li, Shou-Chao Du, Sun-Jun Hu, Shi-Min Chang, Ying-Qi Zhang

**Affiliations:** ^1^ Department of Orthopaedic Surgery, Yangpu Hospital, School of Medicine, Tongji University, Shanghai, China; ^2^ Department of Orthopedics, Tongji Hospital, School of Medicine, Tongji University, Shanghai, China

**Keywords:** femoral neck fracture, in-out-in, femoral neck safe zone, screw placement strategy, insertion angle

## Abstract

**Objective:**

The inverted triangle configuration of the three cannulated screws is the classic fixation method most commonly performed for undisplaced femoral neck fractures in young and geriatric patients. However, the posterosuperior screw has a high incidence of cortical breach, known as an in-out-in (IOI) screw. In this study, we present a novel posterosuperior screw placement strategy to prevent the screw from becoming IOI.

**Methods:**

Using computed tomography data and image-processing software, 91 undisplaced femoral neck fractures were reconstructed. The anteroposterior (AP), lateral, and axial radiographs were simulated. To simulate the intraoperative screw placement process, participants used three screw insertion angles (0°, 10°, and 20°) to place the screw on the AP and lateral views of the radiograph according to the three established strategies. On the AP radiograph, a screw was placed abutting (strategy 1), 3.25 mm away from (strategy 2), or 6.5 mm away from (strategy 3) the superior border of the femoral neck. On the lateral radiograph, all the screws were placed abutting the posterior border of the femoral neck. Axial radiographs were used to evaluate the screw position.

**Results:**

In strategy 1, all the placed screws were IOI regardless of the screw insertion angle. In strategy 2, 48.3% (44/91) of IOI screws occurred at a 0° screw insertion angle, 41.7% (38/91) of IOI screws occurred at a 10° screw insertion angle, and 42.9% (39/91) of IOI screws occurred at a 20° screw insertion angle situation. In strategy 3, no IOI screw occurred, and the screw insertion angles did not affect the safety and accuracy of screw placement.

**Conclusions:**

Screws placed according to strategy 3 are safe. The reliability of this screw placement strategy is unaffected by a screw insertion angle of less than 20 degrees.

## Introduction

Percutaneous cannulated screw fixation is the most popular fixation method for femoral head preservation in young and geriatric patients with undisplaced femoral neck fractures. The inverted triangle configuration of the three cannulated screws is widely used and has been shown to have biomechanical advantages over other configurations (1). However, there has been some concern about the safety of the posterosuperior screw. Hoffmann et al. (2) researched simulated surgeries performed on specimens and reported that 70% of posterosuperior screws were in-out-in (IOI). Yuan et al. (3) retrospectively reviewed the computed tomography (CT) scans of 107 hips after three cannulated screw fixations of femoral neck fractures and identified that 58% of posterosuperior screws were IOI. The IOI posterosuperior screw may pinch or chafe the lateral epiphyseal vessels and subsequently cause avascular necrosis of the femoral head (2).

In clinical practice, surgeons usually position and adjust the femoral neck screws under femoral AP and lateral C-arm fluoroscopy guidance. However, conventional standard anteroposterior (AP) and lateral radiographs are insensitive in assessing IOI screws (4). Multiangle fluoroscopy is frequently used to increase the detection rate of IOI screws, but it may increase the risk of radiation exposure and has a limited indication for adjustment of the nonideal screw position. In this study, we presented a simple and safe strategy for accurate posterosuperior screw placement under AP and lateral radiograph guidance, which has no additional risk of radiation exposure.

## Materials and methods

### Population

From September 2018 to September 2021, pelvis computed tomography (CT) data of 91 consecutive patients with undisplaced femoral neck fractures treated in our hospital were collected. The baseline characteristics of the 91 patients are presented in Supplementary Table S1 (Supplementary Table S1). This study relied solely on CT data and did not involve patients directly. All radiograph simulations and screw placement simulations were completed using Mimics 20.0 software (Materialise Inc., Belgium).

### Simulating radiographs

**Figure 1:**
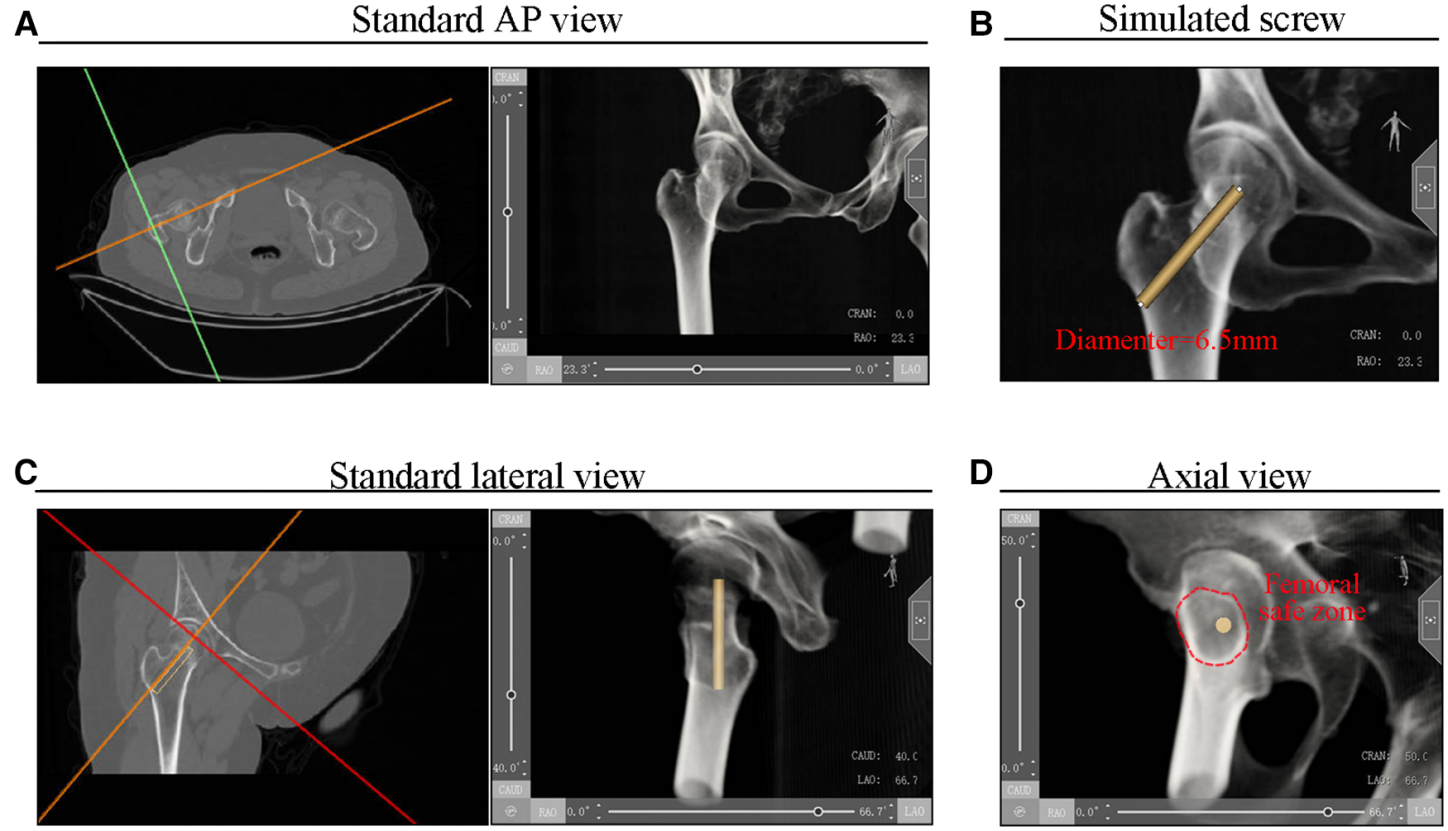
Simulated (**A**) AP, (**B**) lateral, and (**C**) axial views of the radiograph.

First, pelvis CT data were imported into Mimics 20.0 software (Materialise Inc., Belgium). To simulate a radiograph of the injured hip, the gray value in Mimics software's Volume Rendering module was adjusted. The intersection positioning line on the cross-section view was then rotated until the transverse line was parallel to the femur's head–neck axis. The standard AP simulated radiograph of the femur was obtained in this state (Figure 1A). To simulate the 6.5-mm cannulated screw, a cylinder with a radius of 3.25 mm was drawn on the AP view of the radiograph (Figure 1B). Then, the intersection positioning line on the sagittal view was rotated until the transverse line was parallel to the head–neck axis, and the standard lateral view of the simulated radiograph of the femoral neck was obtained (Figure 1C). In this view, the axis of the femoral neck was in line with the axis of the femoral shaft. Finally, based on the lateral view, the CAUD/CRAN degree was further adjusted, and an axial view was obtained in which the cortex of the femoral neck formed a projection along the long axis of the cylinder (Figure 1D). The femoral neck safety zone is defined as the area enclosed by the cortical projection of the femoral neck on the axial view. The simulated screw does not penetrate the cortex if it is placed in this zone. The screw becomes IOI if it extends beyond this area (5).

### Three screw insertion angles

**Figure 2:**
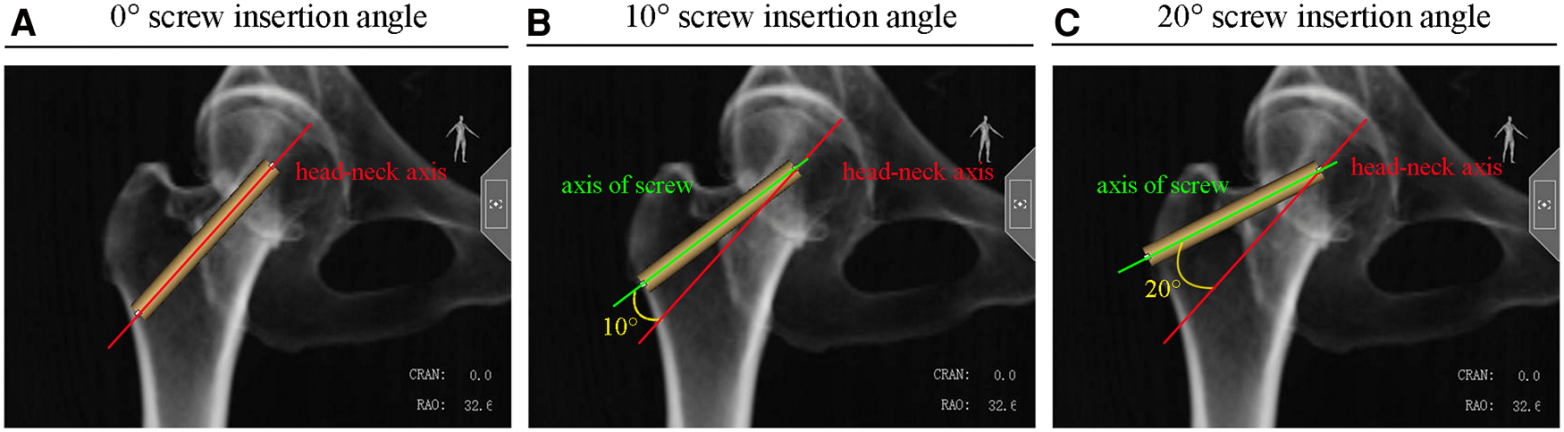
Three insertion angles of the screw including (**A**) 0°, (**B**) 10°, and (**C**) 20°.

In the clinical setting, screws are typically inserted into the femur along but not exactly parallel to the head–neck axis. We simulated three screw insertion angles: 0°, 10°, and 20°. A 0° insertion angle is defined as the screw axis parallel to the head–neck axis (Figure 2A). Screw angles of 10° or 20° to the head–neck axis are defined as 10° insertion angles (Figure 2B) or 20° insertion angles (Figure 2C), respectively.

### Simulating screw placement strategies

**Figure 3:**
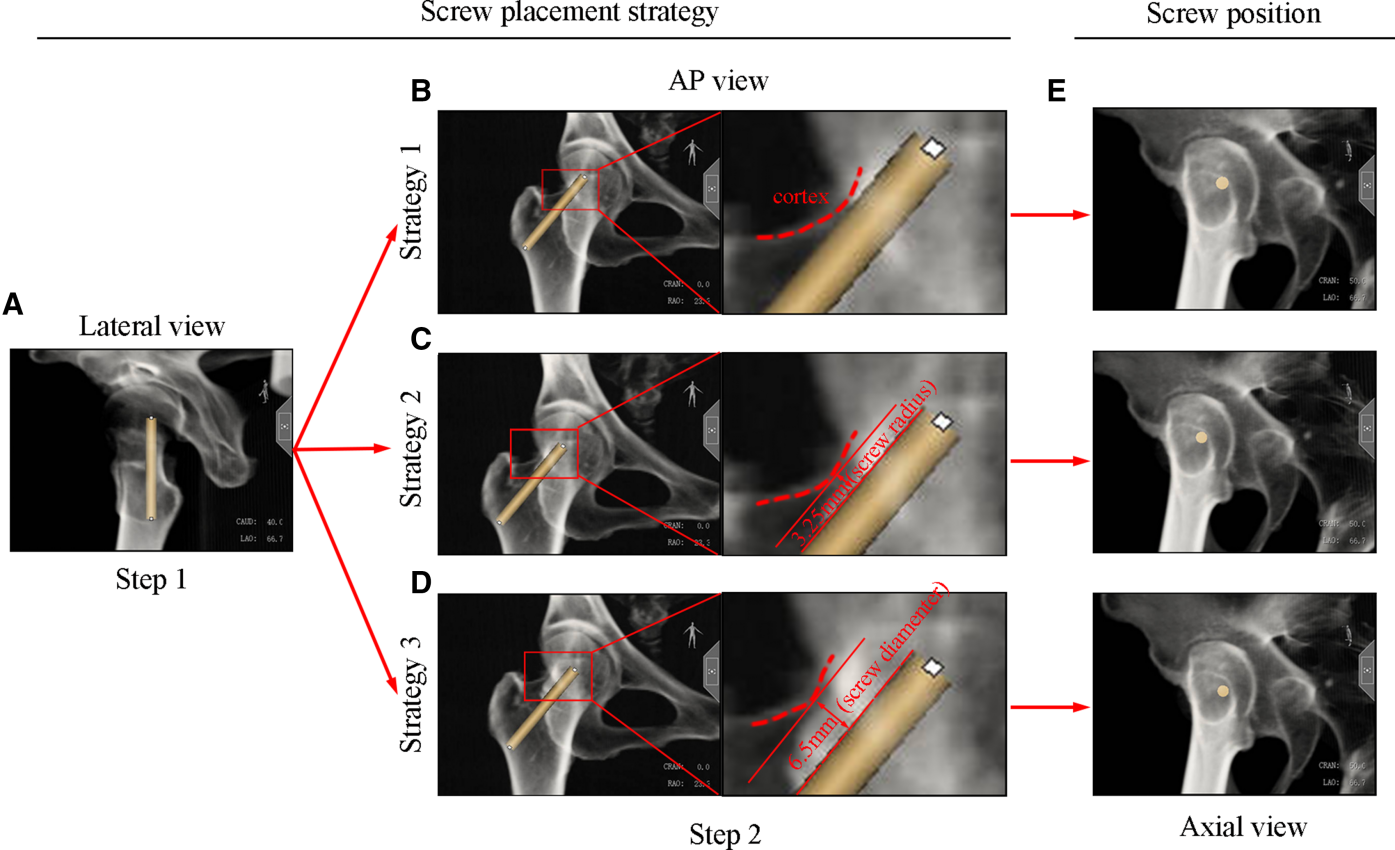
Three screw placement strategies and corresponding axis radiographs.

Screws were placed by two independent participants. Each participant was instructed to use three insertion angles (0°, 10°, and 20°) to place the screw on the AP and lateral views of the radiograph according to the three strategies listed below. All the three screw placement strategies were two-step placement strategies. Step one is to place the screw in the lateral radiograph, and step two is to adjust the screw position in the AP radiograph. On the lateral radiograph, the screw was all placed abutting the posterior border of the femoral neck in all three strategies (Figure 3A). On the AP radiograph, in strategy 1, the screw was placed abutting the superior border of the femoral neck (Figure 3B); in strategy 2, the screw was placed 3.25 mm (screw radius) away from the superior border of the femoral neck (Figure 3C); and in strategy 3, the screw was positioned 6.5 mm (screw diameter) away from the superior border of the femoral neck (Figure 3D). To be consistent with the actual operation, all participants placed the screws on the AP and lateral views by visually assessing under the established strategies without additional ancillary tools. The corresponding axial view was employed to assess the safety of the simulated screw by two independent reviewers (Figure 3E).

### Evaluation of the posterosuperior screw position

**Figure 4:**
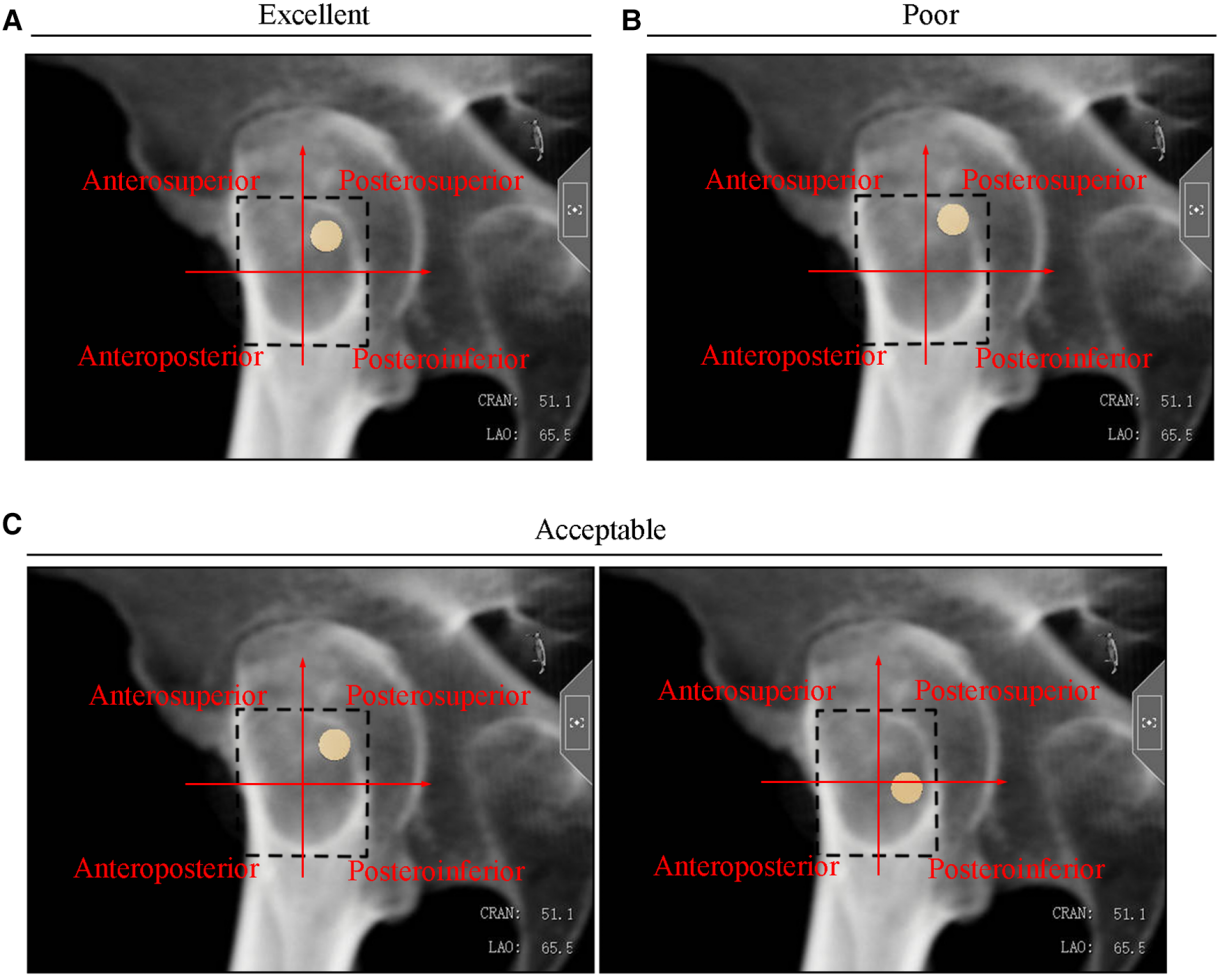
Typical (**A**) excellent, (**B**) poor, and (**C**) acceptable screw positions in the axial radiograph.

The circumscribed rectangle of the femoral neck safety zone was drawn on the axial view. The center of the circumscribed rectangle divided the femoral neck safety zone into four quadrants (anterosuperior, posterosuperior, posteroinferior, and anteroinferior quadrants). The screw position was classified as excellent, acceptable, or poor based on the quadrant location and the cortical invasion. The simulated screw, which is located on the posterosuperior quadrant within 3 mm of the femoral neck cortex and has no cortex invasion, was classified as excellent (Figure 4A). Simulated screws located in the posterosuperior quadrant but extending beyond the femoral safety zone (IOI screw) were classified as poor (Figure 4B). Screws in the posterosuperior quadrant with partial cortical invasion but no cortex penetration were considered acceptable (Figure 4C). Screws with noncortex invasion in the posteroinferior quadrant were also acceptable (Figure 4C).

### Statistical analysis

Continuous variables are presented as the mean ± standard deviation. Categorical variables are presented as a total number plus percentage and were compared using the chi-squared test. All the screws were placed, and their corresponding positions were performed and evaluated by two independent reviewers. The assistance of a third reviewer judged the controversial categorical data. The interobserver reproducibility of screw placement strategies was assessed using Kappa coefficients. All screw placement strategies had Kappa coefficients greater than 0.75 (Supplementary Table S2). The repeatability of all screw placement strategies in this study was good. SPSS 22.0 was used for all statistical analyses, and a *P* value < 0.05 indicated statistical significance.

## Results

Strategy 1 was an undesirable strategy for posterosuperior screw placement, resulting in 100% of the screws becoming IOI regardless of the screw insertion angle. Strategy 2 was also unreliable for screw placement, with 48.3% (44/91) of IOI screws occurring at a 0° screw insertion angle, 41.7% (38/91) of IOI screws occurring at a 10° screw insertion angle, and 42.9% (39/91) of IOI screws occurring at 20° screw insertion angle. There were no significant differences in screw placement among the three screw insertion angles under strategy 1 or 2. For screw placements at an insertion angle within 20°, strategy 3 is a reliable strategy for posterosuperior screw placement and for preventing it from being positioned IOI, with 95.6% of screws inserted at a 0° angle being excellent, 86.8% of screws inserted at a 10° angle being excellent, and 91.2% of screws inserted at a 20° angle being excellent. Under strategy 3, there were no significant differences in screw placement among the different screw insertion angles (*P* = 0.1236) (Table 1).

**Table 1:** Evaluation of screw placement strategies.

Screw placement strategy	Screw insertion angle			*P*-value
	0°	10°	20°	
**Strategy 1**
Excellent	0	0	0	1
Acceptable	0	0	0	
Poor	91 (100%)	91 (100%)	91 (100%)	
**Strategy 2**
Excellent	17 (18.7%)	14 (15.4%)	14 (15.4%)	0.685
Acceptable	30 (33%)	39 (42.9%)	38 (41.7%)	
Poor	44 (48.3%)	38 (41.7%)	39 (42.9%)	
**Strategy 3**
Excellent	87 (95.6%)	79 (86.8%)	83 (91.2%)	0.1236
Acceptable	4 (4.4%)	12 (13.2%)	8 (8.8%)	
Poor	0	0	0	

## Discussion

Previous studies showed that the larger the cross-sectional area of the screw configuration, the better the stability of the internal fixation and the lower the risk of fracture nonunion (6, 7). Thus, the inverted triangle screw configuration must be as dispersed as possible when cannulated screws are used to treat femoral neck fractures. To achieve cortical support for fixating screws in femoral neck fractures, Lindequist et al. (8) suggested that screws be placed within 3 mm of the femoral neck cortex. However, posterosuperior screws were reported to have a high rate of IOI. Although there is no specific report on poor outcomes associated with IOI screws in the literature, vascular lesions of the femoral neck and subsequent avascular necrosis are concerning. How can the posterosuperior screw be placed as close as possible to the posterosuperior femoral neck cortex while preventing the screw from penetrating the cortex? According to the morphology of the femoral neck, some researchers have suggested their ideal configuration for femoral neck screws to prevent them from becoming IOI (9, 10). These ideal screw configurations are based on CT reconstructions, which are difficult to create during traditional surgery under fluoroscopy guidance. John et al. (11) suggested using the piriformis fossa radiographic landmark to avoid IOI posterosuperior screws. However, many piriformis fossae cannot be recognized on the AP view of fluoroscopy images. Screws, while capable of avoiding becoming IOI as suggested by John et al. (11), are frequently placed on the posteroinferior quadrant of the femoral safe zone, deviating from the principle of a scattered inverted triangle configuration (Supplementary Figure S1). In this study, we showed that a posterosuperior screw placed on one screw diameter away from the superior border of the femoral neck on the AP view and abutting the posterior femoral neck border on the lateral view could avoid becoming IOI and be placed in an excellent position. Moreover, the screw insertion angles within 20° rarely affected the accuracy of this screw placement method.

Although computed navigation techniques have advantages in accurate screw placement, conventional fluoroscopy is still the most common ancillary technique for percutaneous cannulated screw placement in femoral neck fracture (12–14). Traditional AP and lateral two-dimensional projections of fluoroscopy provide a cuboidal view of the femoral neck region. However, the femoral neck is an irregular cylinder with native torsion. As a result, the actual femoral neck region was smaller than that represented by fluoroscopy. There is a particular IOI danger zone on the posterosuperior outside the femoral neck. Although a posterosuperior screw in this dangerous zone was visible inside the femoral neck on both AP and lateral radiographs, it actually perforated the cortex. Zhang et al. (5) delineated the fluoroscopic margins vs. the actual femoral neck zone on the axial projection view of 24 femoral necks and indicated that the posterosuperior cortical perforation danger zone accounted for approximately 6.7% of the fluoroscopic IOI safe zone on the femoral neck axial view. Kumar et al. (4) quantitatively measured the safety zone of the femoral neck and pointed out that AP and lateral radiographic margins are not reliably trusted for fixating screws in femoral neck fractures. The results of our study were consistent with their suggestions. According to our findings, the position of the posterosuperior screw abutting the femoral neck border on both the AP and lateral radiographs indicates that it is IOI.

Concerning the AP and lateral radiographs that were insensitive to identifying the IOI screws, researchers are attempting to improve the safety assessment of the posterosuperior screw by using additional oblique radiographs. Terhune et al. (15) recommended −15 roll fluoroscopy in the lateral view to improve the identification of IOI posterosuperior screws. Aibinder et al. (16) suggested that the sequential fluoroscopic rollover images after placement of the posterosuperior guide pin effectively detect IOI position. Although these methods improve IOI detection, there is no clear guidance for screw placement. Through the oblique views of radiographs, surgeons are barely able to determine in which direction the IOI guide pin should be adjusted and at what distance. In our study, we pointed out the screw placement targets on the AP view and lateral radiographs. The obvious ideal position of the posterosuperior screw on the AP and lateral radiographs may encourage surgeons to reduce drilling attempts and radiation exposure during the surgery while achieving a dispersed screw configuration and increasing cross-sectional neck area coverage, thus obtaining cortical support for the fixated screws. Some researchers have encouraged the use of 4.5-mm posterosuperior screws to avoid the repositioning of the screw to IOI in female patients (17). Our strategy allows using 6.5-mm posterosuperior screws to effectively avoid the screw repositioning to IOI and achieve adequate fixation.

The limitation of this study was that patients with displaced femoral neck fractures with inadequate reduction were not evaluated. Posteromedial communications and malreduction also have an impact on screw placement accuracy. All of the CT data used in this study were obtained from Asians. Whether this screw placement strategy would be suitable for other ethnicities requires further investigation. In conclusion, to avoid the screw becoming IOI and to achieve cortical support, the posterosuperior screw should be placed more than 6.5 mm (diameter of the screw) away from the superior border of the femoral neck on the AP radiographs and abutting the posterior border of the femoral neck on the lateral view, and within a screw insertion angle of 20°, which rarely affected the accuracy of this strategy. Using our strategy, AP and lateral radiographs were sufficient in guiding and evaluating screw placement in patients with femoral neck fractures.

## Data Availability

The original contributions presented in the study are included in the article/**Supplementary Material**, further inquiries can be directed to the corresponding author/s.

## Supplementary material

**Figure S1** | Using John's strategy, screw was always placed on the posteroinferior quadrant of femoral safe zone.
