# Supplementary figures and images for: A new posterosuperior screw placement strategy to avoid in-out-in screws in femoral neck fractures
Source: Front Surg. 2023 Mar 17;10:1142135. doi: 10.3389/fsurg.2023.1142135 (PMC10069476; doi:10.3389/fsurg.2023.1142135)

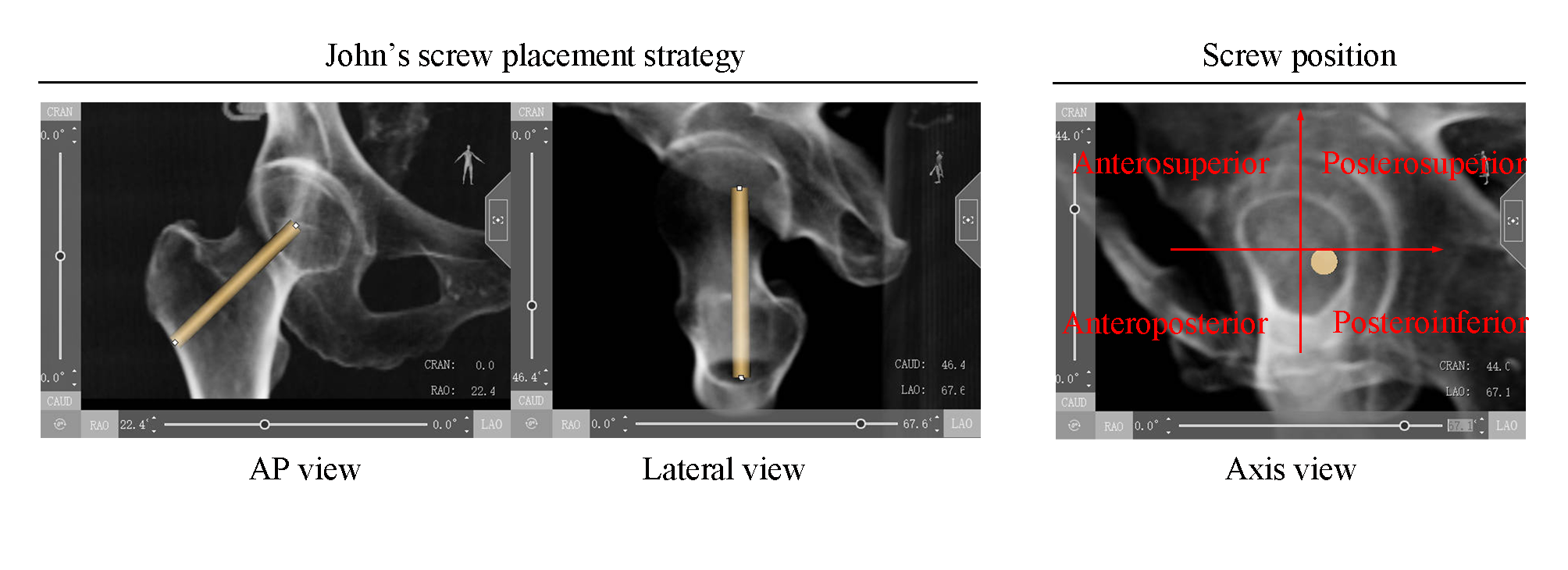

Supplement: Supplementary file 1 [file Image1.tif]
