# Supplementary material for: A new posterosuperior screw placement strategy to avoid in-out-in screws in femoral neck fractures
Source: Front Surg. 2023 Mar 17;10:1142135. doi: 10.3389/fsurg.2023.1142135 (PMC10069476; doi:10.3389/fsurg.2023.1142135)
Supplement: Supplementary file 2 [file Datasheet1.docx]

**Supplemental**

**Supplemental results**

There is a total of 91 patients with 91 undisplaced femoral neck fractures of preoperative CT data included in this study. The mean age of patients was 67.1±8.9. Among the 91 patients, 58.2% (53) were female, 41.8% (38) were male of which 42 hips on the right side and 49 hips on the left side were injured. The mean BMI of the 91 patients was 24.1± 2.1 (Table S1).

Table S1. The clinical characteristics of the 91 patients with undisplaced femoral neck fractures

| Age | 67.1±8.9 |
| --- | --- |
| Sex(female/male) | 53/38 |
| Left/Right | 49/42 |
| BMI | 24.1±2.1 |

Table S2. The inter-observer agreement of screw placement strategies.

| Screw placement strategy | Screw insertion angle | | |
| --- | --- | --- | --- |
|  | 0° | 10° | 20° |
| Strategy 1,kappa | 1 | 1 | 1 |
| Strategy 2,kappa(95%CI) | 0.88（0.79-0.96） | 0.93（0.86-1） | 0.89（0.81-0.98） |
| Strategy 3,kappa(95%CI) | 0.88（0.66-1） | 0.95（0.86-1） | 0.75（0.52-0.99） |

CI: confidence interval
